# Detection of Gastroesophageal Reflux Associated With Apnea in a Preterm Infant Using Multichannel Intraluminal Impedance-pH Monitoring: A Case Report

**DOI:** 10.7759/cureus.77504

**Published:** 2025-01-15

**Authors:** Yuka Hirakuni, Masanori Inoue, Katsuhiro Ogawa, Tomoki Maeda, Kenji Ihara

**Affiliations:** 1 Department of Pediatrics, Faculty of Medicine, Oita University, Oita, JPN; 2 Department of Gastroenterological and Pediatric Surgery, Faculty of Medicine, Oita University, Oita, JPN

**Keywords:** apnea, gastroesophageal reflux, infant, multichannel intraluminal impedance-ph monitoring, proximal esophagus

## Abstract

Gastroesophageal reflux (GER) is a common condition in preterm infants and has been implicated in the development of apnea, although the relationship between the two remains unclear. We report a 29-week gestation male infant who experienced an apnea episode associated with reflux of gastric contents into the cervical esophagus, as identified by multichannel intraluminal impedance-pH monitoring (MII-pH). MII-pH revealed that the apnea episode occurred during a GER event involving prolonged reflux into the cervical esophagus. Interventions, including body positioning, pharmacologic treatments, and dietary adjustments, successfully reduced the frequency of apnea and eliminated episodes of prolonged reflux into the cervical esophagus. This case highlights the utility of MII-pH in identifying reflux characteristics and their potential association with apnea, providing valuable insights for managing similar conditions in preterm infants.

## Introduction

Gastroesophageal reflux (GER) is typically characterized by the movement of stomach contents into the esophagus. It is frequently observed in preterm infants, largely attributed to reduced tone at the gastroesophageal junction [1]. One of the complications associated with GER is apnea, which is thought to occur secondary to vagal nerve stimulation in the posterior pharynx or as a protective mechanism to prevent aspiration [2]. GER is also seen in most preterm infants in varying degrees, with minimal to no effect on the infant [3,4].

While traditional pH probe study is the gold standard for diagnosing GER in infants and children, this method cannot detect nonacidic reflux in frequently fed milk newborns. Multichannel intraluminal impedance-pH monitoring (MII-pH) has recently been employed in GER research [5,6]. The MII measures electrical impedance changes and can track the movement of fluid and air in the esophagus, regardless of the pH and composition of the contents. MII-pH is especially suitable for newborns, identifying more reflux events than traditional pH-metry [4]. However, clinical studies investigating the correlation between GER and apnea in infants using MII-pH are limited, with no evidence linking apnea to GER episodes [3,7].

Apnea of prematurity is a common developmental complication in preterm infants and is primarily attributed to the immaturity of the systems that regulate respiration [8]. It is defined as a cessation of breathing lasting 20 seconds or longer or a shorter respiratory pause accompanied by bradycardia, cyanosis, or oxygen desaturation in infants born before 35 weeks of gestation [8]. This condition is typically categorized into three types: central apnea, arising from the immature brainstem's inability to initiate or sustain adequate respiratory effort; obstructive apnea, resulting from the collapse or blockage of the underdeveloped upper airway despite ongoing respiratory effort; and mixed apnea, characterized by features of both central and obstructive mechanisms [9]. Most apneic events in preterm infants are mixed, characterized by central apnea initiated by airflow obstruction or the reverse scenario [8]. Management strategies for apnea of prematurity in Japan emphasize supportive care, including maintaining an optimal body temperature, ensuring proper positioning to keep the airway open, and applying continuous positive airway pressure (CPAP) to prevent airway collapse [8,10]. For persistent apnea that is not resolved with these measures, pharmacologic therapy with methylxanthines, particularly caffeine citrate, is preferred due to its superior safety and efficacy profiles [8].

Although GER is considered to be associated with apnea in preterm infants, the exact nature of this relationship remains unclear. This may be due to the lack of precise diagnostic methods to distinguish between GER episodes that trigger apnea and those that do not. Given the potential involvement of the laryngeal chemoreflex response, it is likely that significant proximal reflux into the cervical esophagus plays a key role in apnea associated with GER. We herein report an infant who experienced apnea episodes in the context of GER, where refluxate reached above the cervical esophagus, as identified by MII-pH.

## Case presentation

A Japanese male infant was born via vaginal delivery at 29 weeks and one day of gestation with a birth weight of 1,275 grams and was admitted to our neonatal intensive care unit. His Apgar scores were 5 and 8 at one and five minutes, respectively. Postnatally, he was diagnosed with neonatal respiratory distress syndrome and received invasive ventilatory support and surfactant therapy. Two days after intubation, the patient was extubated to nasal CPAP for respiratory support, and enteral feeding was initiated.

Following extubation, episodes of apnea accompanied by bradycardia and oxygen desaturation were detected using continuous transcutaneous oxygen and heart rate monitoring. On the same day, treatment with caffeine citrate was initiated with a loading dose of 20 mg/kg, followed by a maintenance dose of 5 mg/kg/day starting the next day. However, episodes of apnea requiring stimulation to resume breathing persisted. Therefore, the caffeine citrate dose was increased to 10 mg/kg/day on day of life (DOL) 4. While the frequency of apnea episodes remained three to five per day, the infant demonstrated good overall clinical status and steady weight gain. To monitor for potential infections or electrolyte abnormalities, weekly complete blood count and biochemistry evaluations revealed no pathological findings. Since no further interventions were considered necessary, the infant was managed conservatively. By DOL 51 (postmenstrual age (PMA) 36 weeks and three days), the frequency of apnea had decreased to one to three episodes per day, and CPAP and caffeine were discontinued. Oral feeding was introduced on DOL 53 (PMA 36 weeks and five days).

At the PMA of 37 weeks, the infant continued to have one to three episodes of apnea daily. These episodes, characterized by sudden cessation of breathing during sleep or wakefulness, were associated with bradycardia (heart rate of 70-80 bpm) and oxygen desaturation (SpO₂ 60-70%) but resolved promptly with stimulation. A head MRI performed on DOL 68 (PMA 37 weeks and six days) to evaluate potential brain injury showed no abnormalities, and continuous monitoring and observation were maintained. On DOL 83 (PMA 41 weeks and 0 days), the infant experienced an unusually severe episode characterized by cyanosis and bradycardia lasting approximately two minutes, during which the infant was unresponsive to stimuli and required bag-valve-mask ventilation. Comprehensive evaluations were performed to investigate potential underlying causes, including a complete blood count, biochemical analysis, and cerebrospinal fluid examination to rule out infection; an electroencephalogram to assess seizures; and echocardiography and electrocardiography to evaluate for cardiac dysfunction. All findings were within normal limits.

**Figure 1:**
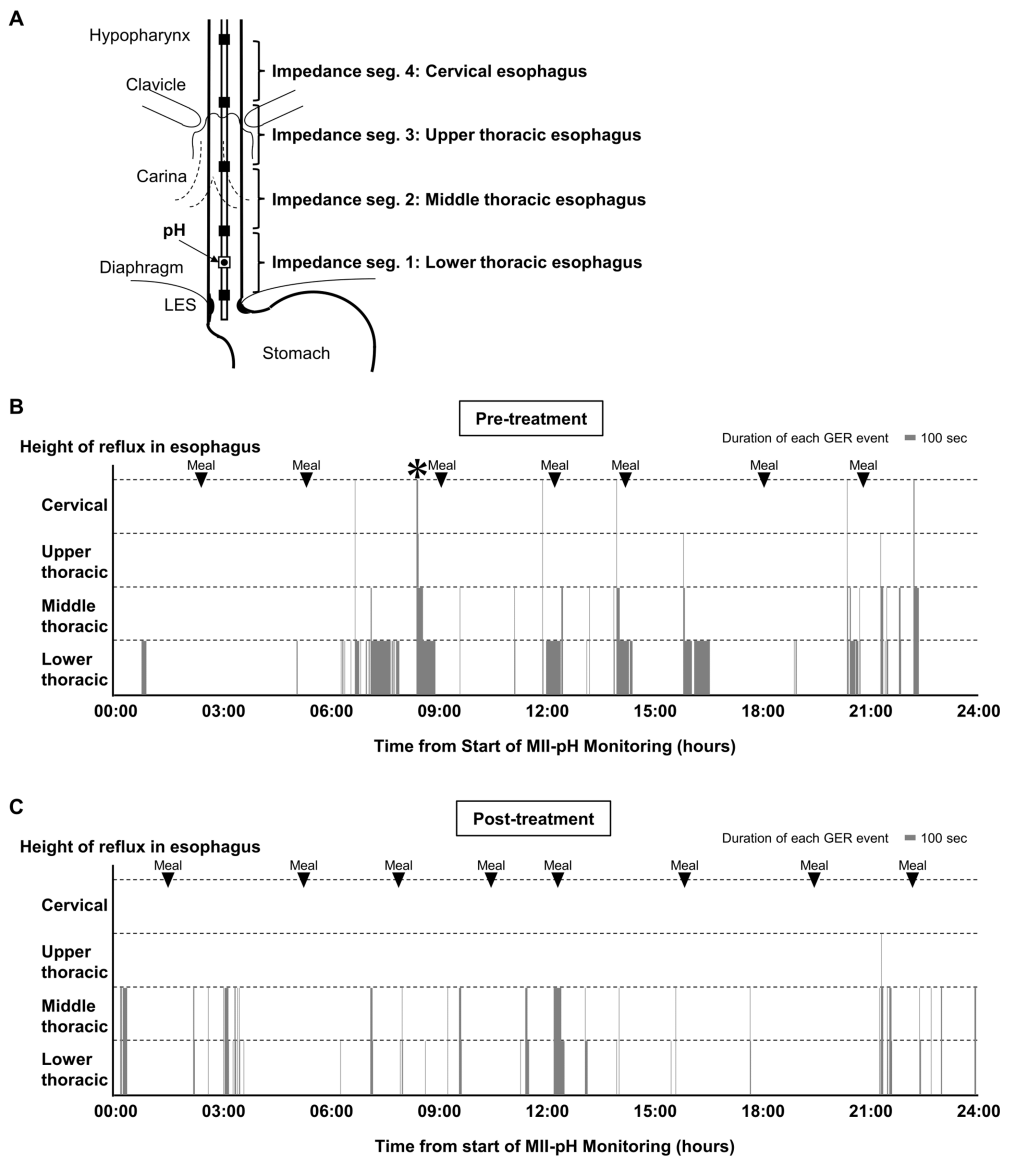
Quantitative evaluation of GER by MII-pH (A) Schematic representation of the recording assembly. The MII-pH catheter had one pH channel within the esophagus and four impedance segments corresponding to each pair of electrodes, recording reflux at the lower, middle, upper thoracic, and cervical esophagus. (B, C) GER events detected by MII-PH. Each bar is an episode of GER. All data from 24-hour records are shown. The vertical axis indicates the height reached by refluxed liquid, and the horizontal axis shows the duration of reflux episodes detected by MII-pH before (B; day 92) and after treatment for GER (C; day 117). The * mark indicates a GER episode accompanied by apnea. GER: gastroesophageal reflux, MII-pH: multichannel intraluminal impedance-pH monitoring Image Credit: Masanori Inoue

To further investigate the cause of apnea, a gastrography was performed on DOL 92 (PMA 42 weeks and two days). The examination revealed the presence of GER, with 30 mL of contrast medium administered via a nasogastric tube, refluxing above the cervical esophagus and associated with episodes of apnea, bradycardia, and SpO₂ reduction. On the same day, an MII-pH catheter (CZPN-BG-5; Sandhill Scientific, Highlands Ranch, CO, USA) was inserted through the nostril and positioned under fluoroscopic guidance, with the pH channel placed in the lower esophagus and the four impedance segments positioned in the lower, middle, upper thoracic, and cervical esophagus (Figure 1A). The MII-pH was conducted using the ZepHr impedance-pH system (Sandhill Scientific, Highlands Ranch, CO, USA), and 40 GER episodes were recorded over 24 hours (Figure 1B). Six GER events involving reflux lasting more than three seconds to the cervical esophagus were identified during this monitoring period. Among these, four episodes lasted three to four seconds, one lasted 10 seconds, and one lasted 15 seconds. Notably, one apnea event occurred during a GER episode in which the reflux of gastric contents persisted for more than 15 seconds to reach the cervical esophagus. We then initiated body positioning intervention with a right-side-down posture and 30° head elevation after feeding, along with the administration of oral rikkunshito (0.2 g/kg/day), famotidine (1 mg/kg/day), and additional dextrin to the breast milk.

Following the abovementioned interventions, the symptomatic apnea episodes reduced to once every three to four days by DOL 107 (PMA 44 weeks and three days), and a repeat MII pH probe on DOL 117 (PMA 45 weeks and six days) detected 35 episodes with no episode lasting >3 seconds in the cervical esophagus (Figure 1C). The infant was discharged home on DOL 119 (PMA 46 weeks and one day) with prescriptions for rikkunshito and famotidine. Discharge instructions included maintaining a 15-degree head-up position for 30 minutes after feeding, and dextrin was discontinued. The infant was followed up at our hospital following discharge. An apnea monitor was used at home until the infant achieved rolling over at six months of age (corrected age of four months), and no further events were observed during this period.

## Discussion

A GER episode with a characteristic record was detected during an apnea event using MII-pH testing. While the total number of GER episodes detected by MII-pH did not change significantly, the episodes in which refluxed milk remained in the cervical esophagus for a prolonged period were no longer observed on repeat examination after introducing interventions. These interventions included body positioning, administration of oral rikkunshito, famotidine, and the addition of dextrin to breast milk. Stimulation of sensory afferent nerve fibers from the superior laryngeal nerve endings in the larynx may induce central apnea [11]. While small amounts of refluxed liquid typically do not stimulate the larynx, fluid infusion into the larynx has been shown to trigger central apnea in infants [12]. This upper airway chemoreflex is more pronounced in preterm infants with immature parasympathetic nervous system development and tends to diminish with age [11]. The treatments addressed prolonged reflux episodes observed in the proximal esophagus during the initial evaluation. Body positioning was employed to facilitate gastric emptying and potentially reduce the likelihood of reflux [13]. Rikkunshito, a traditional Japanese herbal medicine with prokinetic effects, was introduced based on its reported efficacy in reducing vomiting and promoting weight gain in infants with GER disease [14]. Additionally, famotidine, a histamine H2-receptor antagonist, was administered to decrease gastric acid secretion, potentially reducing irritation caused by acidic reflux. Antisecretory treatments, including H2-receptor antagonists, have been reported to alleviate extraesophageal symptoms associated with GER [15]. Dextrin, a thickening agent, was added to breast milk, as thickening agents have been shown to significantly reduce the incidence of prolonged acid reflux episodes [16]. However, it is crucial to emphasize that the observed improvement in reflux patterns during repeat testing may be due to the natural maturation of the lower esophageal sphincter (LES) rather than the interventions themselves. While body positioning, rikkunshito, famotidine, and dextrin were introduced to address prolonged reflux episodes, evidence supporting their efficacy in treating symptomatic GER or preventing GER-associated apnea remains limited [1]. This highlights the need for cautious interpretation of the observed outcomes and underscores the importance of further research.

Preterm and young infants are predisposed to increased reflux of gastric and duodenal contents into the proximal esophagus due to a lower tone of the LES, which places them at increased risk [17]. Critical defense mechanisms, such as esophageal peristalsis, bicarbonate secretion in the esophageal mucosa, and the tone of the lower and upper esophageal sphincters, are underdeveloped in this population [7]. Additionally, frequent feeding with milk, combined with postprandial gastric distension and transient LES relaxation, further exacerbates the high incidence of GER affecting the proximal esophagus during this developmental period [18]. In infants fed milk multiple times daily, weakly acidic or non-acidic gastric contents often reflux into the proximal esophagus [19]. Pharyngeal pH monitoring is a method to detect reflux into the proximal esophagus or pharynx. However, its utility is limited because it can only detect acidic reflux and cannot identify weakly acidic or non-acidic reflux episodes [18]. MII-pH has been introduced to address this limitation and is widely used in the study of GER in infants [7]. Although studies using MII-pH have been conducted in preterm infants, MII-pH profiles focusing on the reflux of gastric contents into the proximal esophagus and its associated symptoms remain underexplored [7,20]. The information provided by MII-pH monitoring - including the extent of reflux reaching the upper gastrointestinal tract, its timing, pH (acidic, weakly acidic, or non-acidic), composition (liquid or gas), and the impact of body position - has the potential to advance our understanding of reflux-related events. Such data could contribute significantly to developing therapeutic strategies for managing infant apnea episodes.

Our report observed apnea episodes occurring when GER reached the proximal esophagus during fluoroscopic examination. During MII-pH monitoring, apnea was observed only during episodes when gastric contents refluxed into the proximal esophagus for prolonged periods. However, due to the nature of this case report and the limited number of apnea episodes recorded during MII-pH monitoring, establishing a definitive causal relationship between reflux reaching the proximal esophagus and apnea in preterm infants remains difficult. Further studies utilizing MII-pH with larger datasets, focusing on the specific locations of reflux within the esophagus, are required to determine the direct correlation between GER episodes and the occurrence of apnea in preterm infants.

## Conclusions

Our case highlights the potential link between GER and apnea in preterm infants, particularly when GER episodes involve the reflux of gastric contents into the cervical esophagus. MII-pH proved to be an effective tool in detecting episodes associated with apnea events. Although a direct causal relationship could not be definitively established due to the limited number of apnea episodes recorded, the findings suggest that when gastric contents reflux into the proximal esophagus, it may trigger apnea, especially in preterm infants with immature gastrointestinal and respiratory systems.
